# Limb Salvage through Intermediary Wound Coverage with Acellular Dermal Matrix Template after Persistent Pseudomonas Aeruginosa Infection in a Burn Patient

**DOI:** 10.3390/ebj3010004

**Published:** 2022-01-12

**Authors:** Mateusz Gładysz, Vincent März, Stefan Ruemke, Evgenii Rubalskii, Peter Maria Vogt, Nicco Krezdorn

**Affiliations:** 1 Burn Center, Department of Plastic, Aesthetic, Hand and Reconstructive Surgery, Hanover Medical School, Carl-Neuberg-Straße 1, 30625 Hannover, Germany; Maerz.Vincent@mh-hannover.de (V.M.); vogt.peter@mh-hannover.de (P.M.V.); krezdorn.nicco@mh-hannover.de (N.K.); 2 Department of Cardiothoracic, Transplantation and Vascular Surgery, Hannover Medical School, Carl-Neuberg-Straße 1, 30625 Hannover, Germany; ruemke.stefan@mh-hannover.de (S.R.); Rubalskii.Evgenii@mh-hannover.de (E.R.); 3 Lower Saxony Centre for Biomedical Engineering, Implant Research and Development, Stadtfelddam 34, 30625 Hannover, Germany

**Keywords:** burn, NovoSorb BTM, pseudomonas, reconstruction, skin graft, skin transplantation

## Abstract

Secondary infections of skin grafts pose a continuous problem in burn patients, very often leading to loss of transplanted skin grafts and making multiple surgical revisions necessary. We present a case report about persisting Pseudomonas aeruginosa infection in burn patients with known diabetes. The burn wounds in lower extremities required repeated debridements, multiple skin grafting attempts and finally an application of the dermal scaffold NovoSorb BTM. With these measures, we managed to undertake a successful reconstruction of infected burn defects and pre-vent an amputation. We concluded that the NovoSorb BTM could be seen as an additional promising tool in a burn surgery armamentarium. In cases where radical surgical wound decontamination is not possible without risking the loss of the limb, the application of NovoSorb BTM over a contaminated field can win extra time for topical infection treatment and additionally provide an excellent skin grafting ground.

## 1. Introduction

Wound infections still pose a significant challenge in the successful treatment of deep thickness burn injuries. A persisting and progressing wound infection can, in the worst-case, result in amputation. Here, we report a 69-year-old diabetic patient who suffered from a deep partial thickness circumferential burn injury of both legs to an extent of 15% of total body surface area (TBSA). After initial therapy and early debridement with immediate skin-graft coverage, we observed a progressive loss of skin graft due to local wound contamination with *Pseudomonas aeruginosa*. After the course of several surgical debridements and topical anti-infective therapy, we were confronted with a deep lower leg defect with uncovered tendons and persisting *Pseudomonas aeruginosa* wound contamination. With a further tissue loss due to the uncontrollable local infection, an amputation seemed unavoidable. We resorted to individual experimental therapy to minimize further tissue loss and ultimately avoid amputation. To control infection and create a viable underground for skin grafting over the exposed tendons, we performed wound coverage with NovoSorb BTM and continued topical infection treatment. After 21 days of the integration phase, we performed a successful skin grafting and were able to transfer the patient with closed wounds to a burn rehabilitation clinic.

## 2. Case Presentation/Treatment Course

**Figure 1:**
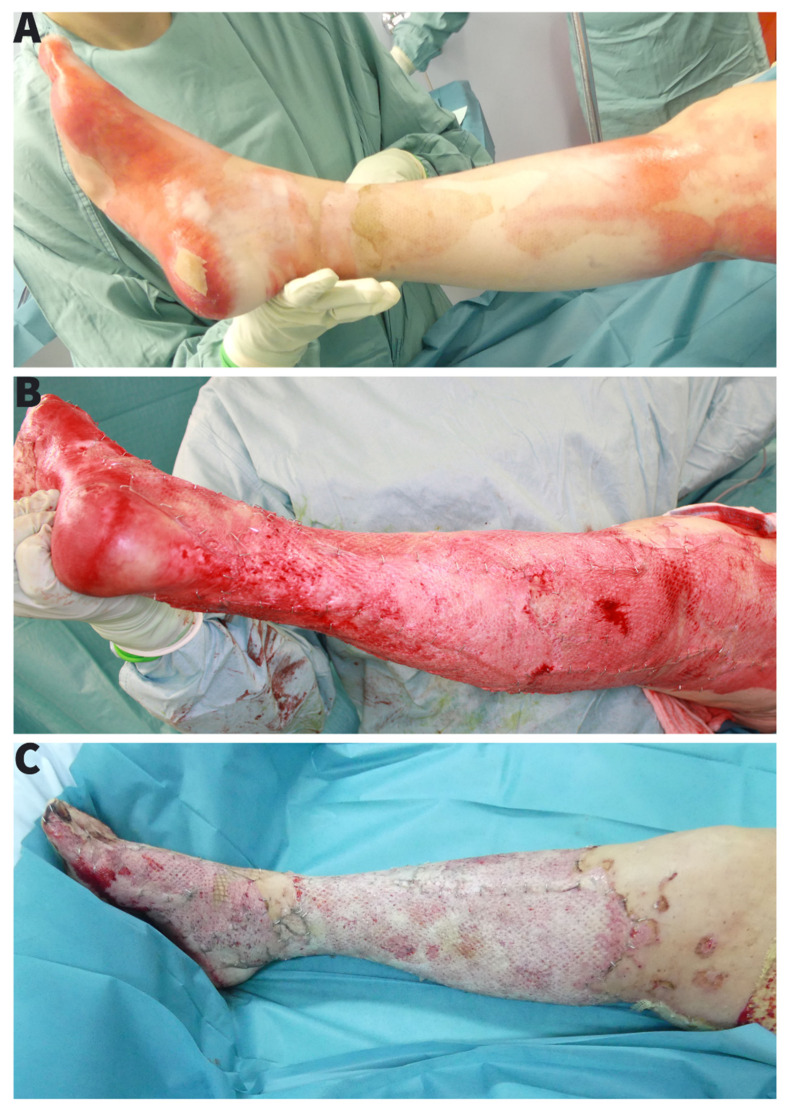
(**A**) Initial trauma with circumferencial deep partial thickness burn injury, (**B**) Directly after first operation with tangentiall necrectomy and split-thickness skin grafting 1:1.5 mesh, (**C**) Skin graft loss after first operation with progressing non-adherence of skin grafts in course of infection.

**Figure 2:**
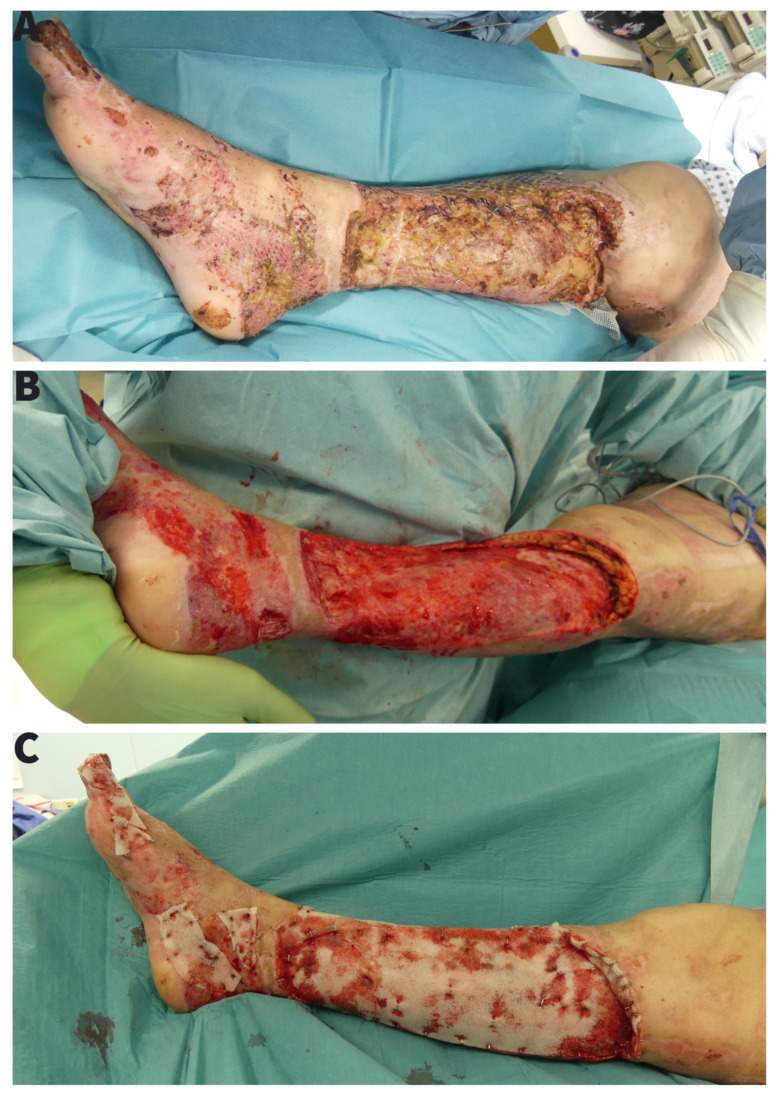
(**A**) Persisting tissue necrosis of the lower leg in course of Pseudomonas aeruginosa infection with loss of the skin grafts, (**B**) Repeated radical epifascial necrectomy with wound coverage with split-thickness skin allograft, (**C**) BTM application after repeated wound debridement and removing of split-skin allografts.

The case presentation is based on operation reports, photo documentation and clinical findings with 1-month follow-up after transfer to a burn rehabilitation clinic. The patient was presented to our burn centre in January 2021 after a domestic burn injury with ethanol. The burning ethanol spilled on the carpet on which the patient was standing and caused deep partial thickness burn of a 15% TBSA of both feet and lower extremities (Figure 1A). After admission, an aseptic washing-lotion (Octenisan^®^ Co., Schülke & Mayr GmbH, Norderstedt, Germany) and a disinfectant (Octenisept^®^ Co., Schülke & Mayr GmbH, Norderstedt, Germany) were used for the initial aseptic and hydrotherapeutic debridement. An escharotomy of the right lower leg with consecutive synthetic skin replacement for temporary wound dressing (EpiGARD^®^ Biovision GmbH, Ilmenau, Germany) was necessary to prevent compartment syndrome. All burn injuries were covered with a polyhexanide hydrogel (Lavanid^®^ wound gel Co., Serag-Wiessner GmbH & Co. KG, Naila, Germany) and fatty gauze (Jelonet^®^ Co., SMITH & NEPHEW, Watford, UK). After assessment of the secondary burns, we performed an early tangential necretomy over the entire burn area with primary skin grafting and standard foam dressing on the third day (Figure 1B). The 0.2 mm skin grafts were taken from thighs bilaterally and meshed 1:1.5. Both legs were immobilized in a cast. The foam dressing was removed on the 5th day. By that time, the microbiological examination (wound swab culture) showed a massive *Pseudomonas aeruginosa* colonization without any prior outside signs of infection. Eventhough we applied diverse topical treatments with topical iodine solution, open wound treatment, or water filtrated infrared light we observed a progressing loss of skin grafts (Figure 1C). A second operation with a radical debridement, including epifascial necrectomy over the right lower leg with repeated autologous skin grafting combined with allogenic skin overgrafting was performed 10 days after the first skin grafting. Unfortunately, after early removal of foam dressing, we observed a persisting wound colonization with *Pseudomonas aeruginosa* and non-adherence of skin grafts (Figure 2A). To treat the infection, we repeated a radical debridement 7 days later, using hydro-surgery with Versa-Jet (Figure 2B). There were no signs of systemic infection therefore none of antibiotics were applied. The wounds were then covered with 1500 cm^2^ of allogenic skin graft (Euro Skin Bank, AJ Beverwijk, Netherlands) and Bactigras (Smith & Nephew GmbH Hamburg, DE) dressing. Topical treatment was extended to include Sulfamylon^®^ (mafenide acetate, USP) as a 5% topical solution. Based on the risk of imminent amputation we sought all possible treatment modalities. As the department for heart and thoracic surgery houses a large bank of a variety of phages—and since we had already successfully applied this as an additional rescue therapy for other devastating wounds—we tested the available database for matching phages and cultivated a specifically matching strain [1]. The method is neither new nor experimental as it has been described already in 1932 and was a standard treatment of wounds until the advent of antibiotics in 1942 [2].

Due to now exposed tendons and persisting *Pseudomonas aeruginosa* infection, the risk of a lower extremity amputation upon further progress was eminent. After minimizing the *Pseudomonas aeruginosa* wound colonization, with measures listed above, we repeated a hydro-surgery debridement with Versa-Jet and covered the wounds with NovoSorb BTM polyurethane based dermal skin template (Figure 2C). The Novosorb BTM was fixated with staplers and incised to provide drainage of wound secretion. We continued an open wound treatment with a daily Sulfamylon^®^ and water-filtrated Infrared light application. By these means, we were able to gain control of the infection and stabilize the wound.

**Figure 3:**
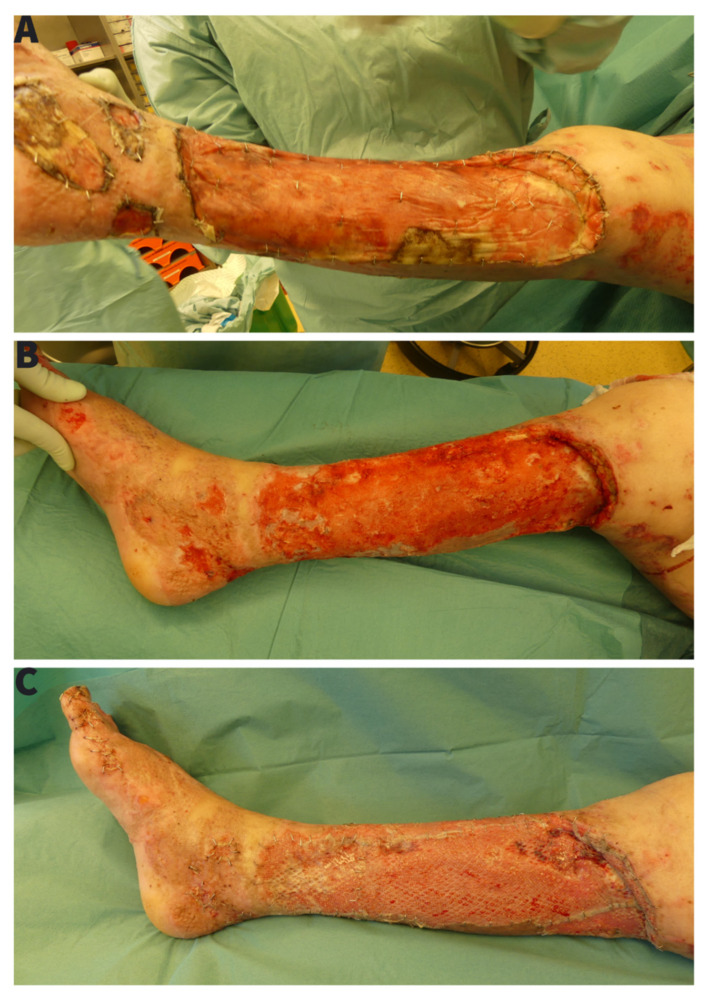
(**A**) 21 days after BTM application with visible almost complete tissue integration and vascularization, (**B**) BTM after seal-removal as a ground for repeated autologous split-skin grafting, (**C**) Skin graft over BTM scaffold after removal of compression garments.

Twenty-one days later (Figure 3B), delamination of the integrated BTM showed a well vascularized wound bed and we could perform a successful autologous skin-grafting (Figure 3C). At the time of skin grafting on BTM, the wound proofed again positive for *Pseudomonas aeruginosa*, so that we decided to remove the foam compression dressing on the 3rd day postoperatively. Skin integration was not affected, as there were no local signs of infection.

**Figure 4:**
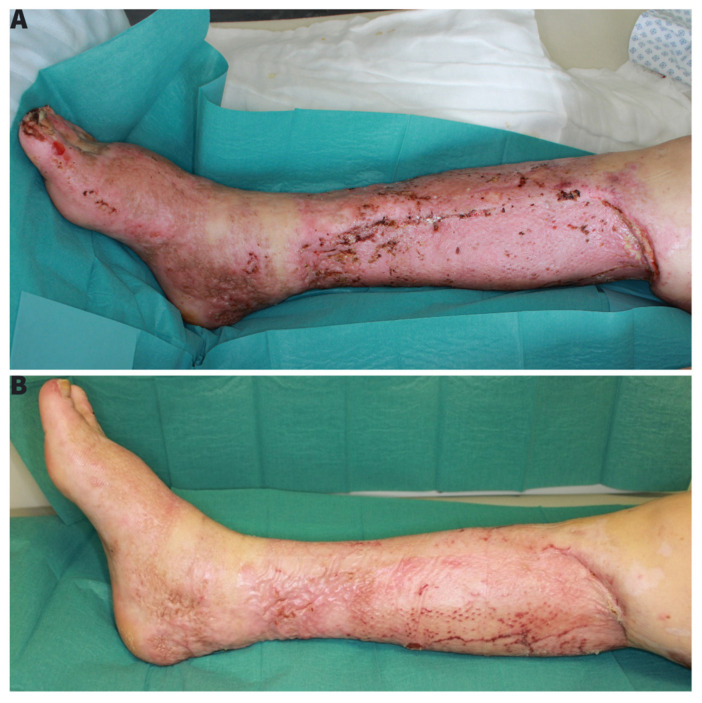
(**A**) End result by transfer to burn-rehabilitation with almost completely consolidated wounds. (**B**) Result after burn-rehabilitation with patient being able to freely walk again.

With successfully integrated skin grafts, the patient could be transferred to the burn rehabilitation clinic (Figure 4A).

## 3. Discussion

Regardless of the advances in the medical field, wound infections still pose a significant challenge in a daily routine of a burn ward [3]. One of the most common types of infections in burn patients is through pseudomonas strains. *Pseudomonas aeruginosa* is a common encapsulated, Gram-negative rod bacterium. It thrives in both aerobic and low-oxygen environments and can be found in soil, water, and skin flora. The ability to aggregate and produce a biofilm only increases its resistance to antimicrobial therapy. In our case, coexisting diabetes is also predisposed to a prolonged infection and poor wound healing. In spite of repeated debridements, we were faced with a persistent infection resulting in a total skin loss putting the patient at risk of a bilateral lower leg amputation. A case series of defect reconstructions using NovoSorb BTM from N. S. Solanki et al. [4] showed a high tolerance of the dermal scaffold against infection. In this case, the wound infection was diagnosed after BTM application. Normally no artificial material is recommended in the infected wound ground. To our knowledge and after a literature search using the keywords “novosorb” and “novosorb BTM” in Pubmed, our case report presents the first application of the NovoSorb BTM in the infected wound with *Pseudomonas aeruginosa* and proved that it could lead to successful treatment.

It is disputable if the same effect could be achieved with more thorough and radical debridement and earlier use of Mafenid. We used primarily topical iodine (Braunol) to treat the skin graft infection according to the standard of care in our clinic.

It is to underline that we used NovoSorb BTM as a temporary measure to improve the wound ground win extra time to gain control over the infection and ultimately allow the successful autologous skin grafting as a definitive wound coverage. We suspect that the neovascularized dermal scaffold BTM as intermediary wound coverage allowed for successful treatment of the *Pseudomonas aeruginosa* infection and subsequent skin grafting [5]. Increased inflammatory response properties of BTM, in comparison to Integra^®^ as shown in the study from P. A. Cheshire et. al, may have helped in the successful treatment [6].

## 4. Conclusions

This case presents a novel reconstructive option in the presence of persisting *Pseudomonas aeruginosa* infection in burn patients. In cases where radical surgical wound decontamination is not possible without risking the loss of the limb, the reduction of contamination and optimization of wound perfusion with bridging therapies is key to enable successful skin grafting, defect reconstruction and limb salvage.
